# Modelling Methionine Requirements of Fast- and Slow-Growing Chinese Yellow-Feathered Chickens during the Starter Phase

**DOI:** 10.3390/ani10030443

**Published:** 2020-03-06

**Authors:** Long Li, K.F.M. Abouelezz, Zhonggang Cheng, A.E.G. Gad-Elkareem, Qiuli Fan, Fayuan Ding, Jun Gao, Shouqun Jiang, Zongyong Jiang

**Affiliations:** 1 College of Animal Science, South China agricultural University, Guangzhou 510642, China; leeloong1985@sina.com; 2 Institute of Animal Science, Guangdong Academy of Agricultural Sciences, State Key Laboratory of Livestock and Poultry Breeding, Key Laboratory of Animal Nutrition and Feed Science in South China, Ministry of Agriculture, Guangdong Public Laboratory of Animal Breeding and Nutrition, Guangdong Key Laboratory of Animal Breeding and Nutrition, Guangzhou 510640, China; abollez@aun.edu.eg (K.F.M.A.); chengzhonggang@yeah.net (Z.C.); fanqiuli_829@163.com (Q.F.); dingfayuan@163.com (F.D.); 3 Department of Poultry Production, Faculty of Agriculture, Assiut University, Assiut 71526, Egypt; alialmaraghy@yahoo.com; 4 Evonik Industries AG Health & Nutrition, Rodenbacher Chaussee, 463457 Hanau-Wolfgang, Germany; jun.gao@evonik.com

**Keywords:** amino acids, nutrient requirements, growth indices, modeling, Chinese yellow-feathered chickens

## Abstract

**Simple Summary:**

In poultry production, consuming diets with low or excessive methionine levels leads to negative effects on growth performance. The requirements of methionine may differ among the fast and slow-growing breeds; therefore, the optimal dietary methionine level should be estimated for each. In this study, six dietary methionine levels were evaluated to estimate the optimal level for fast and slow-growing yellow feathered chicken breeds. The quadratic polynomial and exponential asymptotic regression showed that the optimal methionine requirements for maximal growth performance were 0.50% and 0.53% in the fast-growing breed, and 0.48% and 0.52% in the slow growing breed.

**Abstract:**

Two experiments were carried out to investigate the dietary methionine requirement for fast and slow-growing Chinese yellow-feathered breeds during the starter phase, based on growth variables and regression models. In Experiment 1, a total of 2880 one-day-old Lingnan chicks (fast growing breed) were used to test the methionine requirement from 1 to 21 days of age for males and females separately. Of each gender, 1440 birds were allocated into 6 dietary methionine levels (0.28%, 0.32%, 0.37%, 0.43%, 0.50% and 0.63%), each with 6 pen replicates of 40 chicks. Experiment 2 had the same design with Guangxi chicks (slow growing breed) from 1 to 30 d of age. Results indicated that significant nonlinear or quadratic responses to increasing dietary methionine levels were observed in body weight, daily gain, feed intake and feed conversion ratio of both breeds. In summary, the quadratic polynomial regression showed that the optimal methionine requirements for maximal growth performance of Lingnan chickens were 0.52–0.58% in males, 0.51% in females, and 0.53% in mixed genders. The corresponding values for Guangxi breed were 0.53% in males by quadratic polynomial regression and 0.43% in females, and 0.48% to 0.49% in mixed sexes by exponential asymptotic models.

## 1. Introduction

With the rapid development of economy in China, the second largest worldwide producer of chicken meat, market demands for nutrient-rich and tasty meat have been in continuous increase, which is boosting the industry of Chinese yellow-feathered chickens. The contribution of such chicken type in production has been growing; 3.7 billion birds annually with more than 30% of whole chicken meat shares in recent years [1]. The indigenous chickens have a recent increase in their commercial importance due to their favorable meat color and flavor, which highlights the need for comprehensive studies to enhance their feeding standards. However, only few researches on their nutrient requirements were conducted up till now [2,3,4,5].

Some essential amino acids are often added as pure to poultry diets to ensure the optimal balance required for poultry in order to maximize the production efficiency. Globally, methionine (Met) is considered to be the first limiting amino acid for poultry fed on typical corn–soybean meal-based diets [6]. Met is an essential amino acid doing numerous vital biological activities in animals’ body [7,8]; it has an important role in protein synthesis in addition to enhancing the antioxidant capacity of the organism via participating as a precursor of glutathione that eliminates the reactive oxygen species in the body cells [9,10]. Additionally, the Met is required for the polyaminase synthesis that mediates cell and nucleus division process, and is considered as the most common donor of methyl group consumed in the DNA methylation process [11,12,13,14]. Met, therefore, is required for ensuring normal growth performance in poultry. The dietary supplementation of Met in poultry is commonly used to fulfil the bird requirement of Met and to achieve rapid correction of any deficiency of some nutrients; Met is a precursor of cysteine, succinyl-CoA, creatine, homocysteine and carnitine. Additionally, optimizing the Met level in poultry diets can contribute in reducing the nitrogen emission of nitrogen into the surrounding environment [13,14].

Chinese yellow-feathered chicken is a general name, which consists of quite a few breeds, mainly referring to Chinese local breeds and the improved breeds. Chinese yellow-feathered chickens grow slowly and are reared for a longer time compared with white-feathered broilers. The Lingnan yellow-feathered chicken breed is classified according to its growth rate to fast (1.47–2.30 kg, marketable at 8–10 weeks), medium (1.00–2.27 kg BW, marketable 9–14 weeks), and slow growing (1.06–1.88 kg BW, marketable 12–25 weeks) [3,15,16]. The Guangxi yellow-feathered chicken is a native, slow growing and light-body type breed with good meat quality in China (1.02–1.6kg BW, marketable 13–22 weeks). The nutrient requirements of broilers differ among the different growth stages, which are divided into three periods in Lingnan yellow-feathered broilers: starter (1–21 days old), grower (22–42 days old), and finisher (>42 days), and four phases in Guangxi yellow feathered broilers: starter (1–30 days), grower (31–60 days), early finisher (61–90 days), and later finisher (>90 days old) phases [15,16].

The Met requirement of feeding standard of chicken (CNY/T33-2004) is mainly for medium-growing yellow-feathered broilers [12]. As the dietary Met requirement for the yellow-feathered meat type chickens has not been fully determined or optimized, the present study aimed to estimate it for males and females of Lingnan (a typical fast-growing yellow feathered) and Guangxi (a slow-growing yellow-feathered) chicken breeds during the starter period.

## 2. Materials and Methods

### 2.1. Birds, Diet and Management

Two experiments were carried out to estimate the Met requirement for Lingnan (Exp1) and Guangxi (Exp2) yellow-feathered chicken breeds following the same experimental design. Before trial, the Met content of the basal diet ingredients used in both experiments was determined by ion-exchange chromatography on an automatic amino acid analyzer (L8800, Hitachi, Japan), according to the procedures described by Xi et al. [17]. In each experiment, a total of 2880 one-day-old chicks (50% males + 50% females) were randomly assigned to 6 dietary Met levels, each contained 12 identical floor pens of 40 birds, of which 6 pens were males and 6 were females (n = 240 birds from each sex/treatment). The average initial body weights (g) were 33.88 ± 0.26, 38.75 ± 0.32, and 36.32 ± 0.28 for male, female and mixed sexes of Lingnan chicks, and 31.50 ± 0.23, 31.02 ± 0.25, and 31.20 ± 0.24 for male, female, and mixed sexes of Guangxi chicks. All birds were housed in one environmentally controlled room with dry wooden shaving flooring, continuous artificial lighting was provided from incandescent lamps, and room temperature was maintained at 30 °C in the 1st week and gradually reduced to 25 °C on the 4th week. All chicks were managed according to the Animal Care and Use Committee of Guangdong Academy of Agricultural Sciences Management Guide (GAASIAS-2016-017). The experimental diets were offered from d 1 to d 21 for the Lingnan breed (Exp1) and from d 1 to d 30 for the Guangxi breed (Exp2). Dietary treatments included a Met unsupplemented corn–soybean meal basal diet (Table 1) and the basal diets supplemented with 0.04%, 0.09%, 0.15%, 0.22%, and 0.35% Met in the form of DL-Met (Evonik Industries AG, Hanau-Wolfgang, Germany); the final dietary Met concentrations were 0.28%, 0.32%, 0.37%, 0.43%, 0.50%, and 0.63%.

Pelleted feed and drinking water were freely available to chicks. The different diets were prepared three weeks prior to the trial to allow time for checking content homogeneity, in terms of dry matter, ash, crude protein, ether extract, crude fiber, and amino acid concentrations.

### 2.2. Growth Performance

Birds were weighed at the beginning (day 1) and end of each experiment to record the initial and final body weights (FBW), which were used to calculate the average daily gain (ADG). Average daily feed intake (ADFI) was measured on a per pen basis for the entire experimental period, and the feed conversion ratio (FCR) was calculated. Mortality was checked daily and dead birds were weighed in order to adjust the feed intake calculations.

### 2.3. Statistical Analysis

Data were subjected to one-way ANOVA using the GLM procedure of SAS (version 9.3, SAS Inst., Cary, NC, USA, 2014). Tukey–Kramer test was used for means comparison, and pairwise comparisons among the means were assessed by Duncan’s multiple-range tests at *p* < 0.05. All data were expressed as means and SEM, derived from ANOVA error mean square. When the main effect was significant (*p* < 0.05), linear and quadratic effects of Met content were determined. For key performance variables, the dietary methionine requirement of the birds was estimated using quadratic polynomial (QP) or exponential asymptotic (EA) models by the NLIN procedure of SAS (SAS Institute, 2014).

QP model:

Y = c + bX + aX^2^ (1)

where a = quadratic coefficient, b = linear coefficient, c = intercept. The requirement of Met was defined as Met = −b/(2 × a).

EA model:

Y = a + b × (1 − EXP (−c × (X − d)))(2)

where a = relative response to the diet containing the lowest Met (deficient diet); b = difference between the minimum and the maximum response obtained with dietary Met; c = curve slope coefficient; d = Met level of the deficient diet). The optimal Met was defined as Met = (-ln (0.05)/c) + d, using 95% of the asymptotic response, since the exponential curve never reaches the asymptotic point [18,19,20]. The suitability of the different models was evaluated by the correlation coefficient (R^2^), Akaike information criteria (AIC) and mean square error values (MSE).

## 3. Results

### 3.1. Growth Performance of Lingnan Broilers Aged 1 to 21 Days (Exp 1)

**Figure 1:**
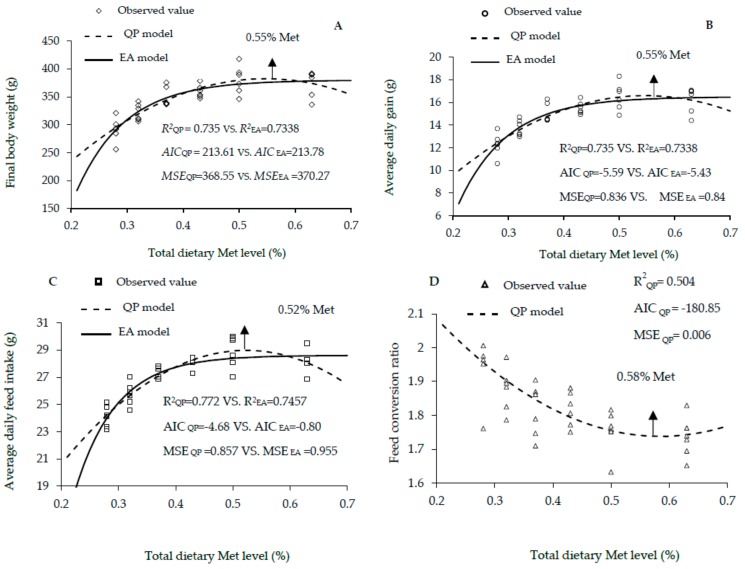
Regression models plot of growth performance as a function of total dietary Met level (0.28, 0.32%, 0.37%, 0.43%, 0.50%, and 0.63% Met) of male rapidly growing yellow-feathered chickens between 0 and 21 days of age. Correlation coefficient (*R^2^*), Akaike information criteria (AIC), and mean squares error (MSE) are indicators for evaluating model fitness. (**A**) The optimum response arrow pointing at 0.55% Met by quadratic polynomial (QP) model. (**B**) The optimum response arrow pointing at 0.55% Met by QP model. (**C**) The optimum response arrow pointing at 0.52% Met by QP model. (**D**) The optimum response arrow pointing at 0.58% Met according to the QP model.

**Figure 2:**
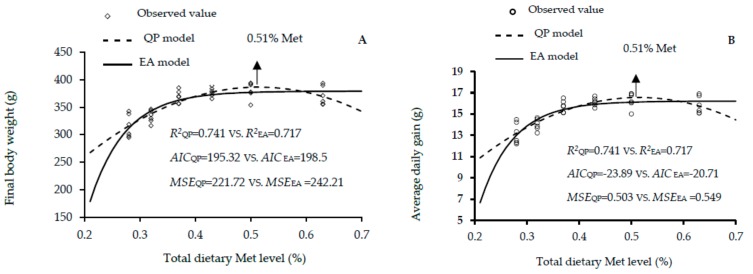

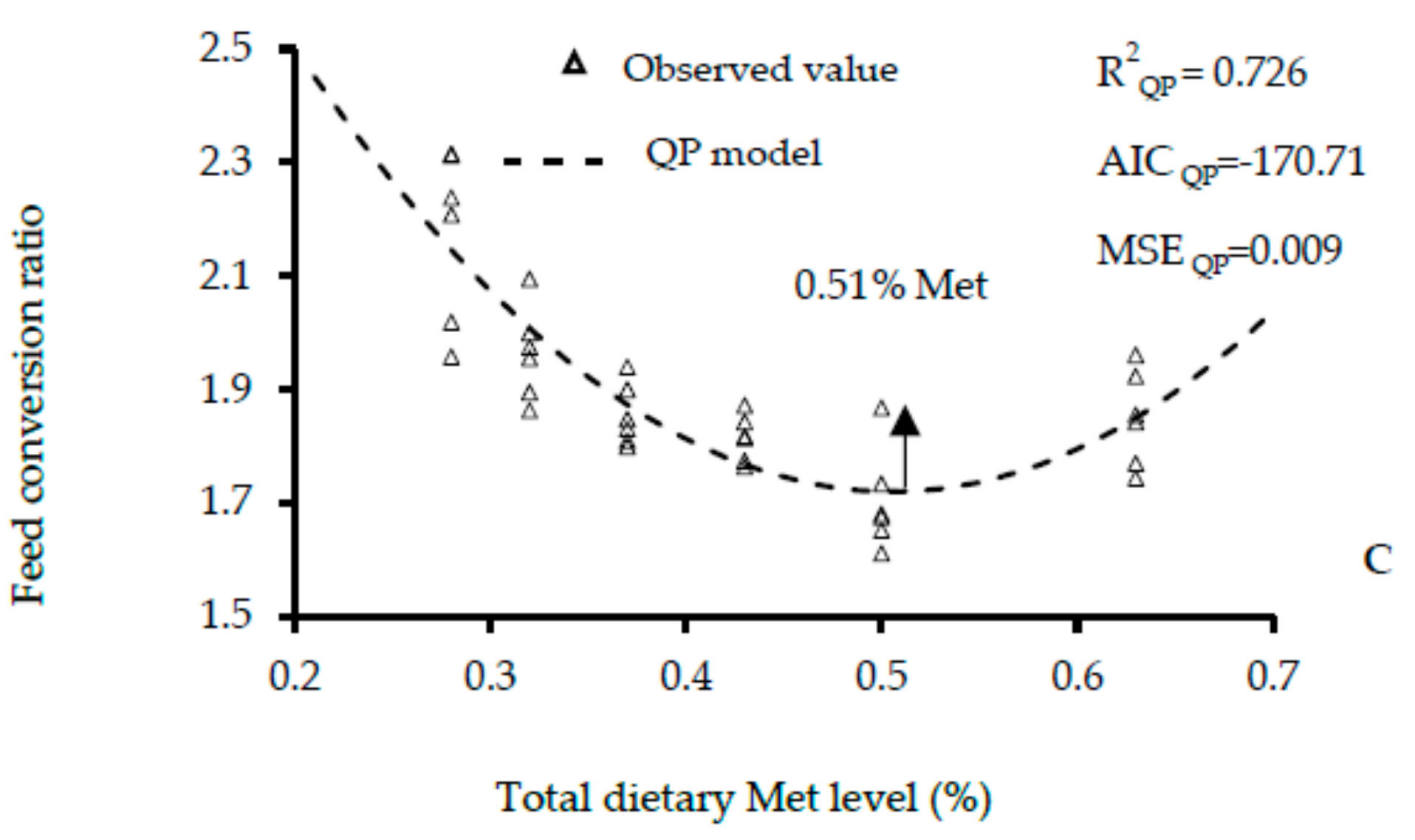
Regression models plot of growth performance as a function of total dietary Met level (0.28%, 0.32%, 0.37%, 0.43%, 0.50%, and 0.63% Met) of female rapidly growing yellow-feathered chickens between 0 and 21 days of age. Correlation coefficient (*R^2^*), Akaike information criteria (AIC), and mean squares error (MSE) are indicators used in evaluating fitness of models. (**A**) The optimum response arrow pointing at 0.51% Met by the best fitting regression model. (**B**) The optimum response arrow pointing at 0.51% Met identified by the best fitting model. (**C**) The optimum response arrow pointing at 0.51% Met.

**Figure 3:**
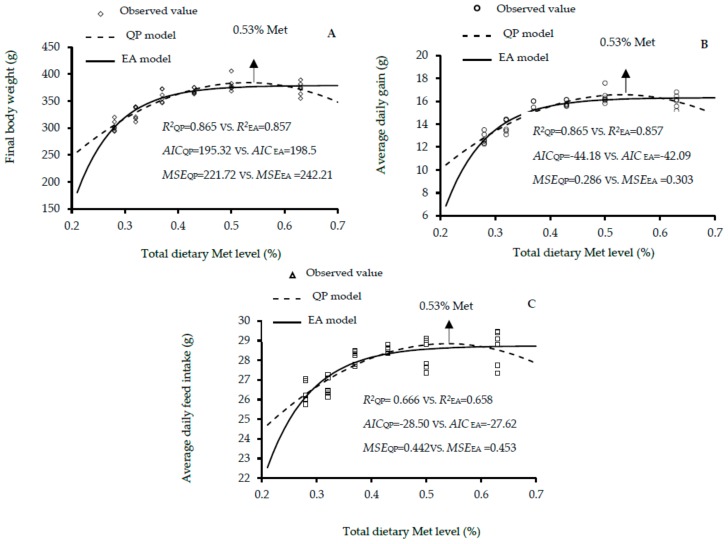
Regression models plot of growth performance as a function of total dietary Met level (0.28%, 0.32%, 0.37%, 0.43%, 0.50%, and 0.63% Met) of mixed sex rapidly growing yellow-feathered chickens aged 0 and 21 days of age. Correlation coefficient (*R^2^*), Akaike information criteria (AIC) and mean squares error (MSE) are used as indicators for evaluation model fitness. (**A**) The optimum response arrow pointing at 0.53% Met according to QP model. (**B**) The optimum response arrow pointing at 0.53% Met by QP model. (**C**) The optimum response arrow pointing at 0.53% Met.

The growth performance traits of male, female and mixed male and female Lingnan yellow-feathered chickens fed different dietary Met levels between 1 and 21 days of age are shown in Table 2, Table 3 and Table 4, respectively. The increase in the dietary Met level showed linear and quadratic effects on the final BW, ADG, ADFI and FCR (linear, *p* < 0.01; quadratic, *p* < 0.01) of males; and final BW, ADG and FCR (linear, *p* < 0.01; quadratic, *p* < 0.01) of females; and final BW, ADG and ADFI (linear, *p* < 0.01; quadratic, *p* < 0.01) of mixed genders. According to the EA and QP regression, the optimal dietary Met level for the highest body weight were 0.54% and 0.55% in males, 0.47% and 0.51% in females, and 0.50% and 0.53% in mixed genders. The corresponding EA and QP values for the highest ADG were 0.54% and 0.55% in males, 0.47% and 0.51% in females, 0.50% and 0.53% in mixed genders. With regard to these results, it is worth mentioning that obtaining the same optimum Met requirement value for maximal final BW and ADG is logically expected; certainly, because these two variables are linearly correlated, where the ADG is calculated as the (final BW—initial BW) /days. This, therefore, led to the same values of R2 and the same calculated Met requirements for these two variables (Table 2, Table 3 and Table 4, Figure 1, Figure 2 and Figure 3). Additionally, the EA and QA models showed that the optimal Met level for ADFI were 0.58% and 0.50% in males, and 0.51% and 0.47% in females, and those of daily feed intake estimated 0.50% and 0.52% in males, and 0.52% and 0.53% in mixed genders. Goodness-of-fit results for the growth performance functions are shown in Figure 1, Figure 2 and Figure 3. The high R^2^, and lowest AIC and MSE values of QP model are shown in Figure 1A–C, Figure 2A,B, Figure 3A–C. The results indicated that the QP model is more adequate for predicting the optimal Met requirements for maximal growth performance in Lingnan broilers, which showed that the optimal Met requirements for Lingnan male, female and mixed sexes were 0.52% to 0.58%, 0.51%, and 0.53%, respectively.

### 3.2. Growth Performance of Guangxi Broilers Aged from 1 to 30 Days (Exp 2)

**Figure 4:**
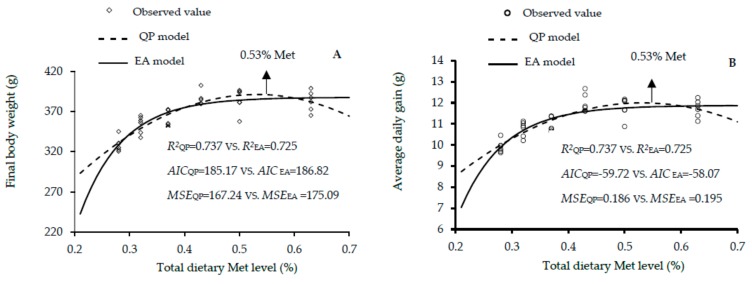
Regression models plot of growth performance as a function of total dietary Met level (0.28%, 0.32%, 0.37%, 0.43%, 0.50%, and 0.63% Met) of male slowly growing yellow-feathered chickens between 0 and 30 days of age. Correlation coefficient (*R^2^*), Akaike information criteria (AIC) and mean squares error (MSE) are indicators used to evaluate fitness of models. (**A**) The optimum response arrow pointing at 0.53% Met by the best fitting model. (**B**) The optimum response arrow pointing at 0.53% Met by the best fitting regression model.

**Figure 5:**
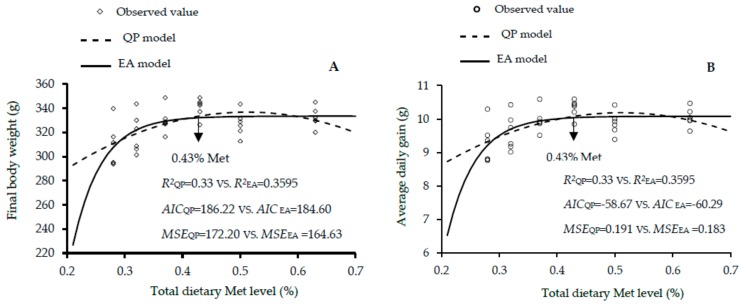
Regression models plot of growth performance as a function of total dietary Met level (0.28%, 0.32%, 0.37%, 0.43%, 0.50%, and 0.63% Met) of female slowly growing yellow-feathered chickens between 0 and 30 days of age. Correlation coefficient (*R^2^*), Akaike information criteria (AIC) and mean squares error (MSE) are indicators used in evaluating fitness of models. (**A**) The optimum response arrow pointing at 0.43% Met by the best fitting model. (**B**) The optimum response arrow pointing at 0.43% Met identified by the best fitting regression model.

**Figure 6:**
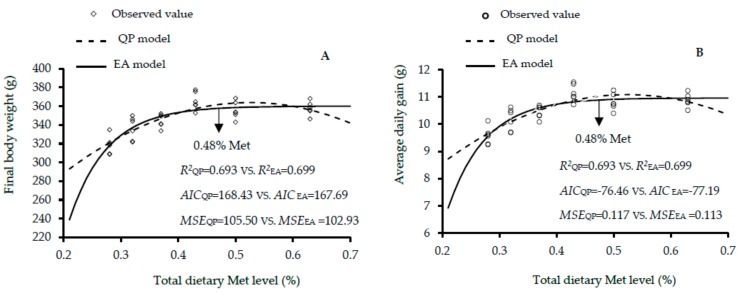

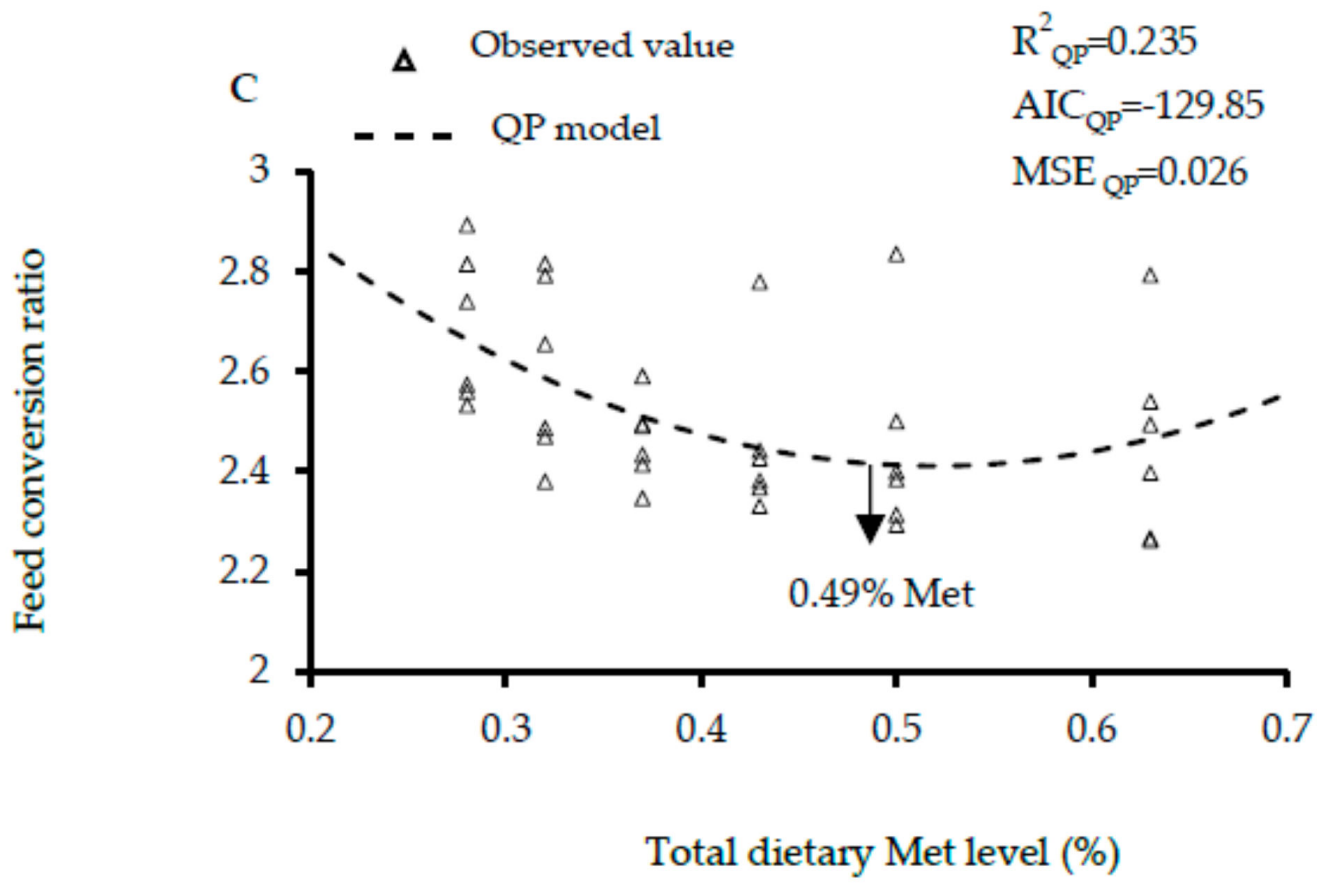
Regression models plot of growth performance as a function of dietary total Met level (0.28%, 0.32%, 0.37%, 0.43%, 0.50%, and 0.63% Met) of mixed sexes of slowly growing yellow-feathered chickens between 0 and 30 days of age. Correlation coefficient (*R^2^*), Akaike information criteria (AIC) and mean squares error (MSE) are indicators of models fitness. (**A**) The optimum response arrow pointing at 0.48% Met by the best fitting model. (**B**) The optimum response arrow pointing at 0.48% Met identified by the best fitting model. (**C**) The optimum response arrow pointing at 0.49% Met.

The growth performance results of male, female and mixed genders of Guangxi yellow-feathered chickens as affected by dietary Met levels between 1 and 30 days of age are shown in Table 5, Table 6 and Table 7. The increase in dietary Met level showed linear and quadratic effects on the final BW (linear and quadratic, *p* < 0.01), ADG (linear and quadratic, *p* < 0.01) and ADFI (linear, *p* < 0.01; quadratic, *p* < 0.05) of males; final BW, ADG, and ADG (linear, *p* < 0.01; quadratic, *p* < 0.01) of females; and final BW (linear, *p* < 0.01; quadratic, *p* < 0.01), ADG (linear, *p* < 0.01; quadratic, *p* < 0.01), and ADFI (linear and quadratic, *p* < 0.05) of mixed genders. According to the EA and QP regression models, the optimal Met for the highest final BW were 0.51% and 0.53 in males, 0.43% and 0.51% in females, and 0.48% and 0.52% in the mixed males and females. The EA and QP indicated that the optimal Met for the maximal ADG were 0.51 and 0.53% in males, 0.43% and 0.51% in females, and 0.48% and 0.52% in mixed genders. Additionally, the QP model showed that 0.49% was optimal for the best FCR in mixed genders. Fitness of the two growth performance models are shown in Figure 4, Figure 5 and Figure 6. QP models showed higher R^2^, lower AIC and MSE values, in estimating the optimal Met requirement (Figure 4a,b and Figure 6a,b) for growth performance of males, whereas the EA model in females and mixed sexes, had higher R^2^, and the lower AIC and MSE values. These results reveal that the QP model are more fitting to predict Met requirement for growing Guangxi males (0–30 days), but the EA function was better fitting for females and mixed sexes. The QP models predicted the requirement of Met for males as 0.53%, and the EA models predicted the requirements of Met for females and mixed genders as 0.43% and 0.48% to 0.49%, respectively.

## 4. Discussion

Diets having low or excessive Met levels could engender important influences on poultry performance [21]. An optimal dietary Met concentration could significantly improve growth performance of broiler chickens when the Met level was lower in the diet [4,5,22,23,24]. The results of the present study confirm that increasing the dietary Met level improved growth performance of Liangnan and Guangxi chicks. This improvement in growth performance is attributable to the important roles of Met in animal’s body, as mentioned in the introduction. Tsiagbe et al. [25] demonstrated that the Met has direct influences on growth performance and immunity in meat-type chickens. In a like manner, Carew et al. [26] reported that Met deficiency reduces the growth and development of lymphoid organs, which have negative effects on growth. The excess of Met, higher than the requirement, is used to cover requirement of some important amino acids such as cystine; two molecules of Met are used in synthesizing one molecule of cystine [27], whereas Met requirement can be covered only by dietary methionine. Additionally, the results here indicated that an excess of dietary Met level did not synchronously improve growth performance of birds and even showed a tendency of negative effects, which was consistent with the findings of Jamroz et al. [28]. Excessive levels of dietary Met can have negative effects on growth. The results of D’Mello and D’Mello [29] indicated that the dietary addition of 20 or 40 g/kg of excess methionine decreased feed intake and reduced body weight gain. Edmonds and Baker [30] found that using an excess of Met at 4% of a corn-soybean meal diet containing 23% protein reduced body weight gain by 92%, whereas similar excesses of lysine, tryptophan, and threonine were far less toxic.

The Met requirement of Chinese yellowed-feathered chicks (unsexed) was 0.46% at the starter phase, according to the old estimations of [16]. According to the results here, however, the Met requirement of Chickens differed between males and females. These results agree with previous findings [4,5,17], which indicated that chicken males and females have different Met requirements. In the present study, the estimated Met requirements of male and female Lingnan, and male Guangxi chickens, determined according to EA and QP models, were higher than the old recommended level (0.46%) of FSC [16]; but the estimated Met requirement of female Guangxi chicken was lower than the recommended value [16]. According to NRC [27], the requirements of Met and Met + Cystine for commercial broiler chickens during the starter period (0–3 weeks) are 0.5% and 0.9% of the diet. The obtained Met requirements here were obtained in the presence of 0.26% cystine (Table 1) in the basal diet. The different Met requirements between breeds (fast and slow growing) or between both sexes is logically attributed to differences in growth rates and genetic potential [31]. Kalinowski et al. [24], reported that Ross 308 broilers optimized final BW (794 g) with dietary Met of 0.50% during 0–21 days; whereas Xi et al. [5], found that the yellow-feathered chicken only needed 0.433–0.435% Met (male) and 0.445–0.454% Met (female) for optimal 21 day BW (male: 351.12 g; female:314.37 g) in the starter phase. According to the available information on the FSC [16] and feeding management regulations of the yellow feathered-chicken [15], the Lingnan chicken breed is classified as a fast-growing breed, and the Guanxi chicken is a slow-growing breed; this can explain the differences in Met requirements obtained here between the two breeds. The different starter phase durations of the Lingnan (0–21 days), and Guangxi (0–30 days) are originally dependent on their growth rate and genetic potential, which have caused the differences in the optimal Met requirement of the two breeds. Similar results were reported by Kalinowski et al. [24] and Dozier et al. [32]. Additionally, several studies indicated that evaluating the response of more than one variable to a dietary nutrient makes it difficult to determine a unique value of the nutrient requirement, i.e., the optimal level of any nutrient required for obtaining the best result of growth rate, body weight, feed intake, feed conversion ratio, meat quality, or immunity indices can differ [2,3,33]; this can explain the different values of optimal Met level for the different growth variables here.

## 5. Conclusions

Supplementation of graded Met levels in basal diets of yellow feathered broilers during the starter period showed beneficial influences on their growth performance indices. The results indicated that the optimal Met requirement differed between the fast (up to 21 d of age) and slow-growing (up to 30 days of age) yellow-feathered chicken breeds as well as between males and females of each breed. The QP regression model was more appropriate for estimating the optimal Met requirements for the best body weight, average daily gain, feed intake and FCR indices of Lingnan (fast-growing) males (0.52% to 0.58%), females (0.51%), and mixed genders (0.53%), as well as for Guangxi (slow-growing) males (0.53%), whereas the EA model was found to be better for estimating the optimal Met requirement of Guangxi females (0.43%) and mixed genders (0.48% to 0.49%).

## Figures and Tables

**Table 1:** Composition of the basal diet (air-dry basis, %).

Ingredient (%)	Amount	Nutrients ^2^	Value
Corn	63.62	AME (MJ/kg)	(12.55)
Soybean meal	20.01	Crude protein (%)	21
Pea protein concentrate	9.68	Lysine (%)	1.2
Soybean oil	2.56	Met ^3^ (%)	0.28
Calcium Carbonate	1.35	Cys ^3^ (%)	0.26
Dicalcium phosphate	1.9	Threonine (%)	0.84
Mineral premix ^1^	0.20	Tryptophan (%)	0.21
Vitamin premix ^1^	0.02	Isoleucine (%)	0.89
Salt	0.49	Valine (%)	0.99
Choline chloride (50%)	0.10	Calcium (%)	1.0
L-Threonine	0.07	Non-phytate phosphorus (%)	0.45
Total	100		

^1^ Totally provided per kg of diet: 1500 IU vitamin A; 200 IU vitamin D3; 10 IU vitamin E; 0.5 mg vitamin K3; 1.8 mg thiamin; 3.6 mg riboflavin; 3.5 mg pyridoxine; 0.01 mg cyanocobalamin; 10 mg pantothenic acid; 30 mg niacin; 0.55 mg folic acid; 0.15 mg biotin; 500 mg choline; 80mg Fe; 8 mg Cu; 80 mg Mn; 60 mg Zn; 0.35 mg I; 0.3 mg Se. ^2^ Values were calculated based on the data provided by Feeding Standard of Chicken (Ministry of Agriculture, China, 2004). ^3^ Analyzed values.

**Table 2:** Effects of total dietary methionine level on growth performance of male Lingnan yellow-feathered chickens aged 1–21 days.

**Variables**	**Total Dietary Methionine Levels (%)**							**SEM ^1^**		***p*-Value**		
	**0.28**		**0.32**	**0.37**	**0.43**	**0.50**	**0.63**			**Met Level**	**Linear**	**Quadratic**
Final body weight (g)	291.67 ^d^		322.44 ^c^	349.26 ^b^	359.79 ^ab^	380.44 ^a^	374.56 ^a^	8.62		<0.0001	<0.0001	<0.0001
Average daily gain (g)	12.28 ^d^		13.74 ^c^	15.02 ^b^	15.52 ^ab^	16.50 ^a^	16.22 ^a^	0.41		<0.0001	<0.0001	<0.0001
Average daily feed intake (g)	24.13 ^d^		25.77 ^c^	27.19 ^b^	28.70 ^a^	28.89 ^a^	28.09 ^ab^	0.47		<0.0001	<0.0001	<0.0001
Feed conversion ratio (g feed: g weight)	1.97 ^a^		1.88 ^b^	1.81 ^bc^	1.82 ^bc^	1.75 ^c^	1.73 ^c^	0.003		<0.0001	<0.0001	<0.0001
**Variables**	**Model**			**Regression Equation ^2^**			**Total** **Dietary Met Level (%)**		**Total** **Daily Met Allowance (g/day)**		***SSR*^3^**	***p*-Value**
Final body weight (g)	Quadratic Polynomial			Y = 15.81 + 1340.52X − 1225.08X^2^			0.55		0.149		12162.24	<0.0001
	Exponential Asymptotic			Y = 291 + 88.99 × (1 − EXP (−11.42 × (X − 0.28)))			0.54		0.146		12218.97	<0.0001
Average daily gain (g)	Quadratic Polynomial			Y = −0.86 + 63.83X − 58.34X^2^			0.55		0.149		27.58	<0.0001
	Exponential Asymptotic			Y = 12.25 + 4.24 × (1 − EXP (−11.42 × (X − 0.28)))			0.54		0.146		27.71	<0.0001
Average daily feed intake (g)	Quadratic Polynomial			Y = 7.04 + 83.86X − 80.13X^2^			0.52		0.141		28.29	<0.0001
	Exponential Asymptotic			Y = 24.03 + 4.58 × (1 − EXP (−13.85 × (X − 0.28)))			0.50		0.135		31.50	<0.0001
Feed conversion ratio				Y = 2.54−2.74X + 2.34X^2^			0.58		0.157		0.212	<0.0001

In the same row, means not sharing a similar superscript (a, b, c, d) differ significantly (*p* < 0.05), the number of replicates was used as the experimental unit (n = 6). ^1^ Standard error of the mean from ANOVA (n = 6). ^2^ Where Y is final body weight, average daily gain, average daily feed intake or feed conversion ratio and X is total dietary content of methionine. ^3^ SSR = sum of squared residuals.

**Table 3:** Effects of total dietary methionine level on growth performance of female Lingnan yellow-feathered chickens aged 1–21 days.

**Variables**	**Total Dietary Methionine Levels (%)**						**SEM ^1^**		***p*-Value**		
	**0.28**	**0.32**	**0.37**	**0.43**	**0.50**	**0.63**			**Met Level**	**Linear**	**Quadratic**
Final body weight (g)	315.34 ^c^	332.84 ^b^	368.9 ^a^	377.26 ^a^	380.81 ^a^	370.91 ^a^	4.73		<0.0001	<0.0001	<0.0001
Average daily gain (g)	13.17 ^c^	14.00 ^b^	15.72 ^a^	16.12 ^a^	16.29 ^a^	15.82 ^a^	0.23		<0.0001	<0.0001	<0.0001
Average daily feed intake (g)	28.53 ^b^	27.46 ^c^	29.13 ^ab^	29.48 ^a^	27.69 ^c^	29.19 ^ab^	0.15		<0.0001	0.147	0.342
Feed conversion ratio (g:g)	2.17 ^a^	1.96 ^b^	1.852 ^bc^	1.83 ^c^	1.70 ^d^	1.85 ^bc^	0.029		<0.0001	<0.0001	<0.0001
**Variables**	**Model**		**Regression Equation ^2^**			**Total Dietary Met Level, %**		**Total** **Daily Met Fed Allowance, g/day**		***SSR ^3^***	***p*-Value**
Final body weight (g)	Quadratic Polynomial		Y = 46.14 + 1323.09X − 1285.88X^2^			0.51		0.145		7316.66	<0.0001
	Exponential Asymptotic		Y = 311.9 + 67.2 × (1 − EXP(−15.64 × (X − 0.28)))			0.47		0.134		7992.86	<0.0001
Average daily gain (g)	Quadratic Polynomial		Y = 0.35 + 63.00X − 61.23X^2^			0.51		0.145		16.59	<0.0001
Feed conversion ratio	Exponential Asymptotic		Y = 13.01 + 3.20 × (1 − EXP(−15.64 × (X − 0.28)))			0.47		0.134		18.12	<0.0001
	Quadratic Polynomial		Y = 3.86 − 8.47X + 8.38X^2^			0.51		0.145		0.281	<0.0001

In the same row, means not sharing a similar superscript (a, b, c, d) differ significantly (*p* < 0.05), the number of replicates was used as the experimental unit (n = 6). ^1^ Standard error of the mean from ANOVA (n = 6). ^2^ Where Y is final body weight, average daily gain, or feed conversion ratio and X is total dietary content of methionine. ^3^ SSR = sum of squared residuals。

**Table 4:** Effects of total dietary methionine level on growth performance of mixed male and female Lingnan yellow-feathered chickens aged 1–21 days.

**Variables**	**Total Dietary Methionine Levels (%)**						**SEM ^1^**		***p*-Value**		
	**0.28**	**0.32**	**0.37**	**0.43**	**0.50**	**0.63**			**Met level**	**Linear**	**Quadratic**
Final body weight (g)	303.50 ^d^	327.65 ^c^	359.10 ^b^	368.53 ^ab^	380.62 ^a^	372.73 ^ab^	3.87		<0.0001	<0.0001	<0.0001
Average daily gain (g)	12.72 ^d^	13.87 ^c^	15.37 ^b^	15.82 ^ab^	16.40 ^a^	16.02 ^ab^	0.18		<0.0001	<0.0001	<0.0001
Average daily feed intake (g)	26.33 ^b^	26.62 ^b^	28.16 ^a^	28.72 ^a^	28.29 ^a^	28.64 ^a^	0.19		<0.0001	<0.0001	<0.0001
Feed conversion ratio (g:g)	2.07 ^a^	1.92 ^b^	1.83 ^c^	1.82 ^c^	1.73 ^d^	1.79 ^cd^	0.017		<0.0001	0.123	0.108
**Variables**	**Model**		**Regression Equation ^2^**			**Total Dietary Met Level, %**		**Total Daily Met Fed Allowance, g/day**		***SSR ^3^***	***p*-Value**
Final body weight (g)	Quadratic Polynomial		Y = 30.97 + 1331.80X − 1255.48X^2^			0.53		0.147		4164.46	<0.0001
	Exponential Asymptotic		Y = 301.3 + 77.56 × (1 − EXP(−13.46 × (X − 0.28)))			0.50		0.139		4413.55	<0.0001
Average daily gain (g)	Quadratic Polynomial		Y = −0.25 + 63.42X − 59.79X^2^			0.53		0.147		9.443	<0.0001
	Exponential Asymptotic		Y = 12.62 + 3.69 × (1 − EXP(−13.46 × (X − 0.28)))			0.50		0.139		10.008	<0.0001
Average daily feed intake (g)	Quadratic Polynomial		Y = 17.71 + 41.35X − 38.39X^2^			0.53		0.147		14.597	<0.0001
	Exponential Asymptotic		Y = 26.14 + 2.59 × (1 − EXP(−12.47 × (X − 0.28)))			0.52		0.145		14.958	<0.0001

In the same row, means not sharing a similar superscript (a, b, c, d) differ significantly (*p* < 0.05), the number of replicates was used as the experimental unit (n = 6). ^1^ Standard error of the mean from ANOVA (n = 6). ^2^ Where Y is final body weight, average daily gain or average daily feed intake and X is total dietary content of methionine. ^3^ SSR = sum of squared residuals.

**Table 5:** Effects of total dietary methionine level on growth performance of male Guangxi yellow-feathered chickens aged 1 to 30 days.

**Variables**	**Total Dietary Methionine Levels (%)**						**SEM ^1^**		***p*-Value**		
	**0.28**	**0.32**	**0.37**	**0.43**	**0.50**	**0.63**			**Met Level**	**Linear**	**Quadratic**
Final body weight (g)	328.96 ^c^	353.96 ^b^	359.07 ^b^	390.74 ^a^	384.15 ^a^	383.04 ^a^	6.18		<0.0001	<0.0001	<0.0001
Average daily gain (g)	9.92 ^c^	10.75 ^b^	10.92 ^b^	11.97 ^a^	11.75 ^a^	11.70 ^a^	0.21		<0.0001	<0.0001	<0.0001
Average daily feed intake (g)	25.76 ^b^	25.82 ^b^	26.37 ^ab^	28.21 ^ab^	27.29 ^ab^	28.84 ^a^	0.82		0.0035	0.003	0.114
Feed conversion ratio (g:g)	2.6	2.41	2.41	2.35	2.32	2.46	0.08		0.1508		
**Variables**	**Model**		**Regression Equation ^2^**			**Total Dietary Met Level (%)**		**Total Daily Met fed Allowance (g/day)**		***SSR*^3^**	***p*-Value**
Final body weight (g)	Quadratic Polynomial		Y = 123.45 + 1007.30X − 947.59X^2^			0.53		0.143		5518.908	<0.0001
	Exponential Asymptotic		Y = 328.50 + 59.13 × (1 − EXP(−12.83 × (X − 0.28)))			0.51		0.138		5777.969	<0.0001
Average daily gain (g)	Quadratic Polynomial		Y = 3.07 + 33.58X − 31.59X^2^			0.53		0.143		6.132	<0.0001
	Exponential Asymptotic		Y = 9.90 + 1.97 × (1 − EXP(−12.83 × (X − 0.28)))			0.51		0.138		6.42	<0.0001

In the same row, means not sharing a similar superscript (a, b, c) differ significantly (*p* < 0.05), the number of replicates was used as the experimental unit (n = 6). ^1^ Standard error of the mean from ANOVA (n = 6). ^2^ Where Y is final body weight or average daily gain and X is total dietary content of methionine. ^3^ SSR = sum of squared residuals.

**Table 6:** Effects of total dietary methionine level on growth performance of female Guangxi yellow-feathered chickens aged 1 to 30 days.

**Variables**	**Total Dietary Methionine levels (%)**						**SEM ^1^**	***p*-Value**		
	**0.28**	**0.32**	**0.37**	**0.43**	**0.50**	**0.63**		**Met Level**	**Linear**	**Quadratic**
Final body weight (g)	308.50 ^c^	318.82 ^bc^	329.90 ^ab^	340.57 ^a^	327.14 ^ab^	332.34 ^ab^	5.61	0.0023	0.011	0.001
Average daily gain (g)	9.25 ^c^	9.59 ^bc^	9.96 ^ab^	10.31 ^a^	9.87 ^ab^	10.04 ^ab^	0.19	0.0023	0.011	0.001
Average daily feed intake (g)	25.57	26.82	24.96	26.31	25.58	24.62	1.11	0.45		
Feed conversion ratio (g:g)	2.77	2.79	2.51	2.55	2.59	2.45	0.11	0.21		
**Variables**	**Model**		**Regression Equation ^2^**			**Total Dietary Met Level, %**	**Total Daily Met Fed Allowance, g/day**		***SSR ^3^***	***p*-Value**
Final body weight (g)	Quadratic Polynomial		Y = 210.40 + 493.91X − 482.58X^2^			0.51	0.131		5682.55	0.001
	Exponential Asymptotic		Y = 307.6 + 25.8 × (1 − EXP(−20.29 × (X − 0.28)))			0.43	0.110		5432.792	0.0006
Average daily gain (g)	Quadratic Polynomial		Y = 5.98 + 16.46X − 16.09X^2^			0.51	0.131		6.314	0.001
	Exponential Asymptotic		Y = 9.22 + 0.86 × (1 − EXP(−20.29 × (X − 0.28)))			0.43	0.110		6.036	0.0006

In the same row, means not sharing a similar superscript (a, b, c) differ significantly (*p* < 0.05), the number of replicates was used as the experimental unit (n = 6). ^1^ Standard error of the mean from ANOVA (n = 6). ^2^ Where Y is final body weight or average daily gain and X is total dietary content of methionine. ^3^ SSR = sum of squared residuals.

**Table 7:** Effects of total dietary methionine level on growth performance of mixed male and female Guangxi yellow-feathered chickens aged 1 to 30 days.

**Variables**	**Total Dietary Methionine Levels (%)**						**SEM ^1^**	***p*-Value**		
	**0.28**	**0.32**	**0.37**	**0.43**	**0.50**	**0.63**		**Met Level**	**Linear**	**Quadratic**
Final body weight (g)	318.73 ^c^	336.39 ^bc^	344.49 ^ab^	365.66 ^a^	355.64 ^ab^	357.69 ^ab^	4.28	<0.0001	<0.0001	<0.0001
Average daily gain (g)	9.58 ^c^	10.17 ^bc^	10.44 ^ab^	11.15 ^a^	10.81 ^ab^	10.88 ^ab^	0.14	<0.0001	<0.0001	<0.0001
Average daily feed intake (g)	25.67	26.32	25.67	27.26	26.44	26.73	0.43	0.2473		
Feed conversion ratio (g:g)	2.68 ^a^	2.60 ^ab^	2.46 ^b^	2.45 ^b^	2.45 ^b^	2.46 ^b^	0.041	0.0105	0.027	0.016
**Variables**	**Model**		**Regression Equation ^2^**			**Total Dietary Met Level, %**	**Total Daily Met Fed Allowance, g/day**		***SSR*^3^**	***p*-Value**
Final body weight (g)	Quadratic Polynomial		Y = 166.93 + 750.60X − 715.09X^2^			0.52	0.137		3466.707	<0.0001
	Exponential Asymptotic		Y = 318.0 + 42.06 × (1 − EXP(−15.14 × (X − 0.28)))			0.48	0.126		3396.603	<0.0001
Average daily gain (g)	Quadratic Polynomial		Y = 4.52 + 25.02X − 23.84X^2^			0.52	0.137		3.852	<0.0001
	Exponential Asymptotic		Y = 9.56 + 1.40 × (1 − EXP(−15.14 × (X − 0.28)))			0.48	0.126		3.774	<0.0001
Feed conversion ratio	Quadratic Polynomial		Y = 3.60 − 4.58X + 4.41X^2^			0.49	0.129		0.874	0.016

In the same row, means not sharing a similar superscript (a, b, c) differ significantly (*p* < 0.05), the number of replicates was used as the experimental unit (n = 6). ^1^ Standard error of the mean from ANOVA (n = 6). ^2^ Where Y is final body weight, average daily gain or feed conversion ratio and X is total dietary content of methionine. ^3^ SSR = sum of squared residuals.
